# Editorial: Advancing critical discovery of novel approaches to understanding and eliminating pain inequities

**DOI:** 10.3389/fpain.2023.1209573

**Published:** 2023-05-11

**Authors:** Staja Q. Booker, Calia A. Morais, Ericka N. Merriwether

**Affiliations:** ^1^ College of Nursing, University of Florida, Gainesville, FL, United States; ^2^ Department of Hematology and Oncology, University of Alabama at Birmingham, Birmingham, AL, United States; ^3^ Department of Physical Therapy, New York University, New York, NY, United States

**Keywords:** pain research, disparities, inequities and inequalities in health, pain, social determinansts of health, pain mechanisms

**Editorial on the Research Topic** Novel approaches to understanding and eliminating pain inequities

Goldberg and McGee posited that chronic pain is a global public health priority (1), and Booker and colleagues added that chronic pain is also a national disparities problem (2) and by acknowledging such, we can begin to [over]stand the multifaceted and interdisciplinary nature of pain as well as the nexus between pain and social determinants of health. The exponential growth of research in pain disparities over the last 20 years unequivocally demonstrates the persistent and glaring racial, ethnic, and social inequities and biases in pain reporting and management that lead to disability and diminished quality of life across pain conditions and patient populations. Despite a strengthened and renewed focus on illuminating major contributors to pain disparities, such as structural and systemic racism and aversive social determinants of health, current research paradigms continually fail to support critical discovery.

*What is “critical discovery”?* Critical discovery uniquely, judiciously, and analytically examines cultural and structural mechanisms of pain inequities, centers the perspectives of people with lived experiences with chronic pain, applies the intersectionality framework in real-time or “realistically”, and develops innovative methodologies to interrogate the impact of social and political determinants of health on pain. The application of such critical and reflective approaches into pain research praxis builds on the scientific approach and incorporates strategies that promote unbiased measurement and reporting of sociocultural and structural factors as well as the inclusion of contextualized and culturally-relevant variables into predictive models of pain outcomes (3). Findings from these inquiries become part of the “scientific canon” to further legitimize and expand this body of critical discovery by maximizing and broadening our arsenal of traditional or innovative designs to ask and answer questions about pain disparities in multiple, hidden, and vulnerable populations (Box 1).

BOX 1 **3 M's to address in pain disparities research.**

To eliminate disparities, inequities, and injustices in pain research, management, and policy, we must address three important types of information:

**MISINFORMATION**: disparities or other research knowledge that have perpetuated fallacies, misrepresented individuals, or contributed to fault finding and blaming/shaming; must correct unfounded beliefs about pain in various groups.

**MISSING INFORMATION**: gaps in [under]standing and knowledge, what are we not asking, unexplored mechanisms and personal/lived experiences, and complexity and “noisiness” of real-life intersectionality.

**MISSED INFORMATION**: hidden narratives, key factors or potentially significant discoveries that have been traditionally overlooked, unrecognized, or [under]stood due to measurement incongruence, error and invariance, the use of inappropriate methodologies, or incorporating stakeholders without cultural allyship.

©2023. Staja Q. Booker, PhD, RN. First quoted in the International Association for the Study of Pain's Black History Month social media posts (February 9, 2023).

Advancing the field of health equity in pain management requires that we approach complex methodological and clinical problems in racialized populations from a justice-based lens. Recently, Mathur et al. argue that “pain disparities are most appropriately conceptualized from an injustice perspective” and presented a dynamic model demonstrating how layers of injustice interact and intersect with multiple pain processes (4). More importantly, we should consider asking questions such as “What factors or mechanisms are contributing to maintaining disparities and inequities in pain?” Reframing our program of research questions may accelerate the transition towards equity in pain management (2, 3).

The primary purpose of this research topic (special issue), “Novel Approaches to Understanding and Eliminating Pain Inequities,” was to present novel research across the translational research spectrum that measured and documented the effect of social factors, culture, and beliefs on pain mechanisms and outcomes. This research topic was conceptualized with the benefit of assessing and harmonizing past discoveries and embracing the emergence of new and important subfields (e.g., sociopolitical pain neuroscience) that specifically address how sociocultural and structural factors, such as racism, shape pain neurobiology and intersect with cultural values and beliefs to influence the incidence, prevalence, and presentation of pain in different cultures and ethnicities across pain conditions.

In this issue, five articles represent a range of topics from epigenetic pain biomarkers to biopsychosocial approaches to pain assessment in Black men. The diversity of the Topic Editors, manuscript reviewers, authors, and content injected novel research methodologies and findings into the scientific canon. The guest editorial team itself had a representation of women from racially underrepresented groups, and reviewed submissions from authors at all career stages and from different nationalities, cultures, and ethnicities.

Our first publication by Aroke et al. examined genome-wide DNA methylation in a cross-sectional study of adults with chronic low back pain. They found that select functional genomic and stress-related pathways were associated with internalized stigma, suggesting that stress-inducing DNA methylation may be an important link between internalized stigma and outcomes in adults with non-specific chronic low back pain. Powell-Roach et al. also investigated the relationships between genetic polymorphisms and clinical and experimental pain in adults with sickle cell disease (SCD) with African ancestry. Results showed that a genotype of the arginine vasopressin receptor 1a gene (AVPR1A), a gene that has been implicated in pain in other populations, was not associated with clinical or experimental pain in this understudied pain population. The third study, led by Walsh et al., investigated lifetime and repeated experiences of ostracism (experiences of being ignored or socially excluded) in participants across six studies. Their analysis revealed that more frequent lifetime experiences of ostracism were associated with laboratory-based measures of pain sensitivity. Further, having a racialized identity significantly moderated the relationship between experiences of lifetime ostracism and multiple measures of pain sensitivity (cold pain threshold, heat pain tolerance, and after-sensations). Elucidating environmental and behavioral factors that maintain or exacerbate pain is a critically important but often neglected area of investigation that was tackled by Mickle et al. Considering environmental and behavioral variables, sociodemographic groups remained a significant predictor of clinical pain, experimental pain, and physical function in adults with or at risk for developing knee osteoarthritis. The critical importance of evaluating and interpreting pain experiences of racialized adults at the intersections of race and gender were highlighted by Baker et al. The group conducted a secondary analysis of data from a randomized, controlled clinical trial of a community-based health intervention in racialized non-Hispanic Black men. Results from the study showed that nearly a quarter of racialized non-Hispanic Black men reported pain in excess of 30 days, and that pain was associated with greater somatization and other select demographic characteristics.

The articles included in this research topic leverage existing sociopolitical pain neuroscience and diversity science frameworks to examine research questions that align with national priorities to address and eliminate inequities in pain management across the lifespan. Further, the research presented positions us to address the need to (A) decolonize terminology and scientific constructs and identify innovative and more specific ways to measure the behavior or outcome of interests and (B) match constructs and processes with salient experiences of the population. In consequence, the neurobiological relationships between the mechanisms of nociception, pain, and sociocultural and structural factors that were once muted or prematurely inconclusive might change if we expand our measures, concepts, and phenomena. Consider these three tenets:

• Social and ecological mechanisms prime and shape pain experiences. For example, social indicators are factors that are amenable when identified early and treated aggressively before they become “determined” or embedded. Both social indicators and social determinants of health (SDoH) influence how pain is experienced.
• Psychological mechanisms impact pain modulation, experience, and perceptions.
• Biological mechanisms underlie the felt experiences of pain processes, and should not be considered in isolation from social, structural, and psychological factors.

The results and conclusions from the studies included in this special issue also highlight opportunities for future studies. This is one of the few special issues in pain science dedicated to exploring and capitalizing on a focus on pain mechanisms and pain inequities while also emphasizing the diversity in research topic, research location, and the teams. The majority of pain research included in this special issue feature racialized non-Hispanic Black/African Americans. We acknowledge that pain disparities are not limited to non-Hispanic Black/African Americans, but also impact other marginalized populations nationally and globally. Moreover, research in pain equity and management often centers on the perspectives of people from Western, industrialized, educated, rich, and democratic (WEIRD) countries. Thus, future studies should examine social and ecological factors in countries in the Global South and other global regions that best reflect the sociocultural and structural mechanisms of pain disparity in these areas, and how they intersect with cultural values and beliefs to influence pain presentation across pain conditions. In addition, the articles in the special issue primarily featured adult populations. We encourage the study of mechanisms and management of pain across the lifespan in different cultural contexts, a transgenerational view particularly in medically underserved and vulnerable populations.

Consider this as the beginning to revolutionize pain disparities and identify justice and parity as our guiding lights toward innovation, progression, and *critical discovery* for pain equity.

## References

[1] Goldberg DS McGee SJ. Pain as a global public health priority. BMC Public Health. (2011) 11:770. http://www.biomedcentral.com/1471-2458/11/770 10.1186/1471-2458-11-770 21978149 PMC3201926

[2] Booker SQ Bartley EJ Powell-Roach K Palit S Morais C Thompson OJ The imperative for racial equality in pain science: a way forward. J Pain. (2021) 22(12):1578–85. 10.1016/j.jpain.2021.06.008 34214701 PMC9133713

[3] Booker SQ Baker TA Esiaka D Minahan JA Engel IJ Banerjee K A historical review of pain disparities research: advancing toward health equity and empowerment. Nurs Outlook. (2023) 71:101965. 10.1016/j.outlook.2023.101965 37023670 PMC11198876

[4] Mathur VA Trost Z Ezenwa MO Sturgeon JA Hood AM. Mechanisms of injustice: what we (do not) know about racialized disparities in pain. Pain. (2022) 163(6):999–1005. 10.1097/j.pain.0000000000002528 34724680 PMC9056583

